# Asymmetric Outcomes of Type 1 Retinopathy of Prematurity after Bilateral Intravitreal Ranibizumab Treatment

**DOI:** 10.1155/2017/1741386

**Published:** 2017-03-29

**Authors:** Qiujing Huang, Qi Zhang, Yu Xu, Xunda Ji, Ping Fei, Jie Peng, Yi-an Li, Peiquan Zhao

**Affiliations:** Department of Ophthalmology, Xin Hua Hospital Affiliated to Shanghai Jiao Tong University School of Medicine, Shanghai, China

## Abstract

*Purpose.* To present cases with retinopathy of prematurity (ROP), who were treated with intravitreal injection of ranibizumab (IVR) and had unpredictable asymmetric outcomes. *Methods.* A retrospective review was performed in infants with type 1 ROP and had bilateral IVR (0.25 mg/0.025 mL) as initial treatment. Patients were classified into the asymmetric outcome group and the symmetric outcome group. *Results.* Eighty-four patients (168 eyes) were included. There were 18 eyes of 9 patients (10.7%) in the asymmetric outcome group and 150 eyes of 75 patients (89.3%) in the symmetric outcome group. In the symmetric outcome group, 86 eyes (57.3%) had ROP regression, 60 eyes (40%) had reactivation requiring laser treatment, and 4 eyes (2.7%) progressed to retinal detachment requiring vitrectomy. In the asymmetric outcome group, one of the eyes of the 9 patients had ROP regression with/without reactivation after IVR, while the contralateral eyes had negative response, including remarkable posterior fibrosis, partial or total retinal detachment, and vitreous hemorrhage. There was statistically significant difference between the birth weight of the two groups. *Conclusion.* Contralateral eyes with ROP can take a different clinical course after ranibizumab treatment. High rate of reactivation after IVR is another concern that ophthalmologists should pay attention to.

## 1. Introduction

Retinopathy of prematurity (ROP) is a retinal vasoproliferative disorder. It continues to be a significant cause of childhood blindness. Laser photocoagulation is the current standard treatment for ROP [1]. Although cryotherapy and laser treatment can cure ROP disease in most cases, they may cause complications such as peripheral visual field defect and myopic shift. Since the role of vascular endothelial growth factor (VEGF) in the pathophysiology of ROP has been well studied, the use of anti-VEGF agents is emerging as a treatment for ROP [2–4]. The only prospective, controlled, randomized, multicenter trial about anti-VEGF treatment for ROP—Bevacizumab Eliminates the Angiogenic Threat of Retinopathy of Prematurity (BEAT-ROP) study—showed that bevacizumab was effective in treating ROP and was more effective than laser treatment in zone 1 ROP cases [5]. On the other hand, reports about reactivation and retinal detachment after injection were not rare [6–8]. What is more, many questions remain unanswered, including the optimal dose and timing of injection, systemic safety, and long-term complications.

In this report, we describe nine cases of type 1 ROP that had asymmetric outcomes after intravitreal injection of ranibizumab (IVR, Lucentis**®**) treatment.

## 2. Materials and Methods

This was a retrospective study which was conducted in the referral ROP screening center in Xin Hua Hospital, affiliated to Shanghai Jiao Tong University School of Medicine. The medical records of patients who were diagnosed with type 1 ROP and had bilateral IVR as initial treatment from January 2012 to December 2014 were reviewed. Patients were classified into asymmetric outcome group (different additional treatments or asymmetric anatomic outcomes in two eyes) and symmetric outcome group. Patients with a follow-up of less than six months were excluded. Each patient's parents or legal guardians were required to sign a consent form before any examination or treatment. This study was approved by the Ethics Committee of Xin Hua Hospital.

Infants were screened if they were born at gestational age of less than 32 weeks or/and their birth weight was less than 2000 g or if they had an unstable clinical course as determined by the infant's neonatologist [9]. Patients' age, gender, family, and birth history, as well as systemic and other ocular anomalies, were noted. Patients were screened by binocular indirect ophthalmoscopy and RetCam (Clarity Medical Systems, Pleasanton, California, USA) fundus photography. Ultrasound examination was given to patients whose fundus was invisible due to corneal opacity or leucocoria. IVR was provided to patients with type 1 ROP. Infants treated with IVR were examined a day after the procedure and weekly thereafter until full vascularization of the retina was observed. If they did not respond positively to the treatment, conventional laser treatment and/or surgery was performed. No second injection of IVR was given to patients. IVR (0.25 mg/0.025 mL), laser treatment, lensectomy, and vitrectomy were performed by the same surgeon (PQZ). Systemic conditions of infants were checked every month after injection by neonatologists.

We performed statistical analysis with the program IBM SPSS 22 (SPSS Inc., Chicago, IL). Continuous variables were summarized as mean and standard deviation (SD) because data were normally distributed. An independent *t*-test was used to compare continuous data between the group with asymmetric outcome and the group with symmetric outcome. *P* value <0.05 was considered statistically significant.

## 3. Results

During the study period, 168 eyes of 84 infants were diagnosed with type 1 ROP and received bilateral IVR as initial treatment. Among them, 18 eyes of 9 patients (10.7%) had asymmetric outcomes in contralateral eyes after IVR (Table 1). The remaining 150 eyes of 75 patients (89.3%) had symmetric outcomes. In the symmetric outcome group, 32 eyes (21.3%) had aggressive posterior retinopathy of prematurity (APROP), 30 eyes (20%) were classified as zone I stage 3+, 16 eyes (10.7%) were classified as zone II stage 3+, 20 eyes (13.3%) were classified as zone I stage 2+, and 52 eyes (34.7%) were classified as zone II stage 2+. In the asymmetric outcome group, the gestational age ranged from 27 to 32 weeks, with a mean of 29.6 ± 1.8 weeks; the birth weight ranged from 980 to 1690 g, with a mean of 1222.2 ± 216.6 g; and the IVR injection time ranged from 34 to 42 weeks postmenstrual age (PMA), with a mean of 37.0 ± 2.4 weeks (Table 2). There was a statistically significant difference between the BW of the asymmetric outcome group and symmetric outcome group. There were no statistically significant differences between the mean GA, PMA, and postnatal age (PNA) at IVR.

In the symmetric outcome group, 86 eyes (57.3%) had ROP regression after IVR, 60 eyes (40%) had reactivation requiring additional laser treatment, and 4 eyes (2.7%) of 2 patients which progressed to retinal detachment required lens-sparing vitrectomy. All eyes had flat retinas at the last visit. The time between reactivation and the initial IVR was 16~108 days, with an average of 56.8 ± 17.1 days. The mean PMA at reactivation was 43.4 ± 3.4 weeks.

In the asymmetric outcome group, one of the eyes of the 9 patients had ROP regression with later reactivation in 8 of them after IVR requiring secondary laser treatment, while the contralateral eyes had negative responses requiring additional surgical treatment, including remarkable posterior fibrosis, partial or total retinal detachment, and vitreous hemorrhage (VH) (Table 1). Retinas were attached in 16 eyes (88.9%) at the last visit. The follow-up period of all eyes ranged from 8 to 30 months, with a mean of 14.4 ± 8.9 months. No noticeable systemic complications related to IVR were observed.

### 3.1. Infant 2

**Figure 1:**
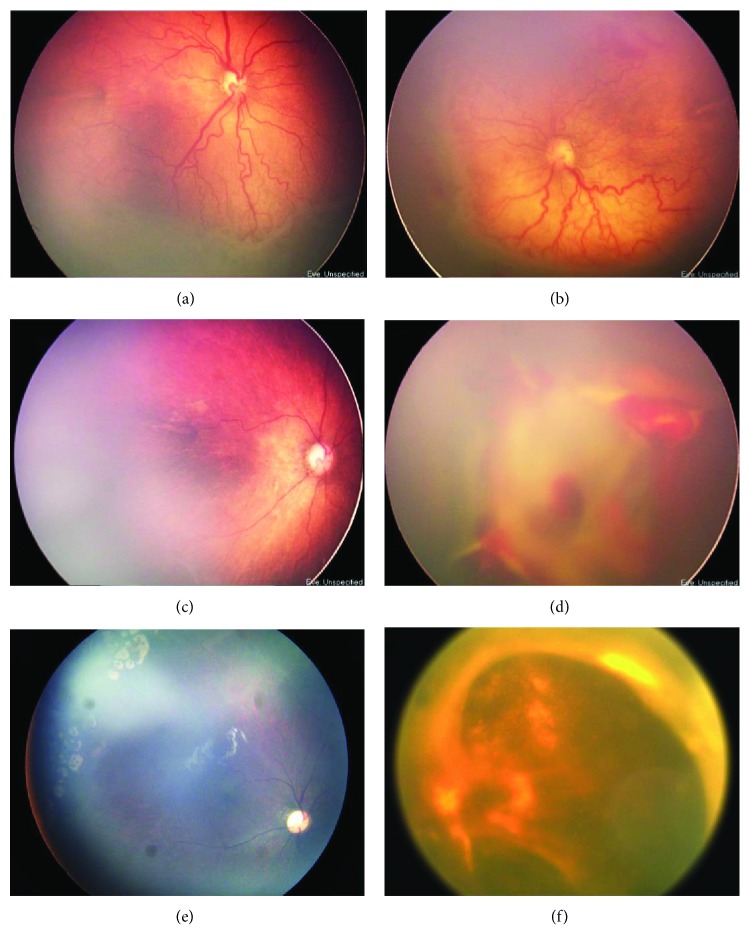
Before treatment, both eyes were diagnosed as stage 3+ ROP in zone I ((a) and (b)). Ten days after IVR, the right eye revealed regression (c), but the left eye revealed stage 5 ROP with marked posterior fibrosis and vitreous hemorrhages (VH) (d). The right eye received laser treatment at 43 weeks PMA, and the retina was flat at the last follow-up (e). The left eye received a lensectomy and vitrectomy at 57 weeks PMA, and the retina was not reattached at the last follow-up (f).

Infant 2 was born at 29 weeks of gestation with a birth weight of 1.2 kg. At 37 weeks PMA, both eyes were diagnosed as stage 3+ ROP in zone I with mild preretinal hemorrhages. The infant received bilateral IVR at 37^+4^ weeks PMA. The right eye regressed first and recurrence occurred in zone II, which was diagnosed as stage 2 ROP without plus at 43 weeks PMA and required laser treatment. Unpredictably, the left eye was diagnosed as stage 5 ROP with marked posterior fibrosis and VH at 39 weeks PMA (10 days post-IVR). The disease rapidly progressed to the shallow anterior chamber and finally received a lensectomy and vitrectomy at 57 weeks PMA. The retina was not reattached (Figure 1).

### 3.2. Infant 7

**Figure 2:**
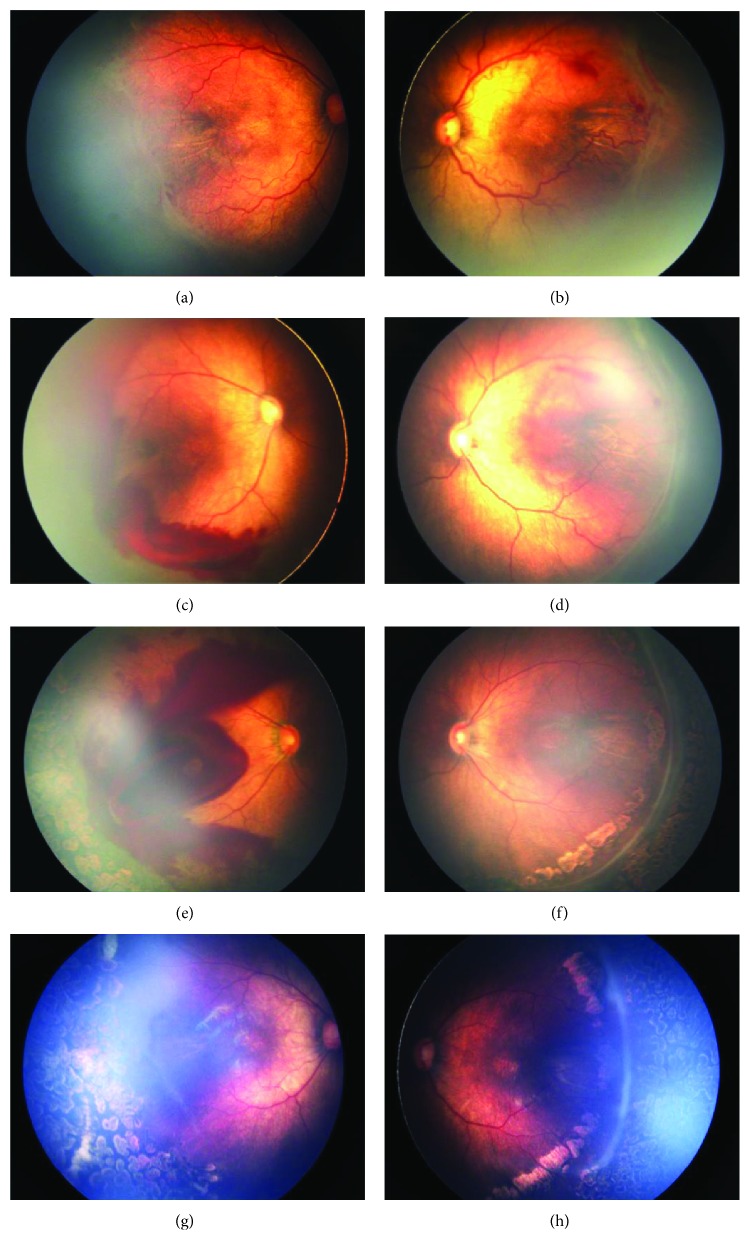
Before treatment, both eyes were diagnosed as stage 3+ ROP in zone I ((a) and (b)). Nine days post-IVR, regression of ROP and plus disease was noted in both eyes, but there were vitreous and preretinal hemorrhages only in the right eye ((c) and (d)). Thirty-eight days post-IVR, hemorrhages in the right eye continued to progress even after laser treatment (e) and covered the macula; and the eye eventually required LSV treatment. One month after LSV treatment, the retina of the right eye was flat with peripheral laser spots, and there was no sign of hemorrhages. The left eye received laser treatment due to persistent zone II avascularity at 43 weeks PMA (f), which was resolved by the last follow-up (h).

Infant 7 was born at 31 weeks of gestation with a birth weight of 1.38 kg. The infant was transferred to our clinic at 41^+6^ weeks PMA with stage 3+ ROP in zone I and received IVR in both eyes at 42 weeks PMA. Regression of ROP was first noted in both eyes, and then severe vitrial and preretinal hemorrhages were noted at 43^+2^ weeks PMA (nine days post-IVR) in the right eye. Hemorrhages continued to progress after laser treatment; and at 47 weeks PMA (38 days post-IVR), hemorrhages covered the macula and the eye, which eventually required LSV treatment. In the left eye, we observed persistent zone II avascularity that required laser treatment (Figure 2).

## 4. Discussion

We described nine cases of ROP that had asymmetric outcomes after bilateral IVR as initial treatment. Similar ROP cases having asymmetric outcomes after intravitreal injection of bevacizumab (IVB) have been reported [8, 10]. However, no such cases have been reported after IVR.

It is difficult to explain why although ranibizumab was administered for both eyes on the same time, but asymmetric outcomes were observed in some patients. The only factor that influenced the outcome in our series was BW. The mean BW of the asymmetric outcome group (1222.2 ± 216.6 g) was smaller than that of the symmetric outcome group (1412.2 ± 335.6 g, *P* = 0.001). We hypothesize that subtle differences in the levels of moieties in the vitreous hemorrhage, such as VEGF, erythropoietin, and insulin-like growth factor-1, [11], may have caused contralateral eyes to follow an asynchronous disease course. Thus, the injection time and dose suitable for one eye was not suitable for the other eye. In infant 2, for instance, RetCam fundus photography revealed that both eyes had zone I, stage 3+ ROP before IVR treatment with the same degree of ridged membrane formation and preretinal hemorrhage; however, at 10 days post-IVR, the left eye revealed a marked extraretinal fibrovascular proliferation (EFP) and VH, while the retina of the right eye was flat with regressed plus disease.

The reactivation rate of ROP after IVR was relatively high. The reported rate of reactivation after IVB was 0~4.3% [5, 12]. Compared with ranibizumab, bevacizumab has a longer half-life [13, 14], it reduces serum VEGF levels more significantly, and systemic VEGF suppression lasts longer [15, 16]. Although this may make ranibizumab a better option for premature patients, it may also translate into a higher chance of reactivation [6]. The reported rate of recurrence with conventional laser therapy was about 26% [5, 17]. Unlike laser treatment destroying the retina, retinal vessels continue to develop after IVR. This can theoretically decrease the supplementary laser spots needed after reactivation and the subsequent destruction of peripheral visual fields, which might offer potential vision benefits [17]. Moreover, the interval from treatment to reactivation of anti-VEGF treatment was longer than that of laser therapy [5]. Approximately ninety percent of infants demonstrated reactivation after IVB within a 10-week window from approximately 45 to 55 weeks of adjusted age [18]. The mean reactivation PMA after IVR in this study was 43.4 ± 3.4 weeks, which was earlier than IVB. Thus, the follow-up examinations after anti-VEGF treatment should last longer than laser treatment [18].

The timing of the administration of anti-VEGF therapy is of utmost importance [19]. The mean injection time in the asymmetric outcome group was later than the treatment of zone I ROP in the BEAT-ROP study (34 ± 1 weeks PMA) [5]. All of our patients received IVR within phase II of ROP [5, 20], and they all had plus disease before the initial treatment. It is possible that an older PMA is a risk factor for complications after IVR treatment.

Anti-VEGF agents might exacerbate preexisting fibrosis and retinal detachment due to traction [21]. In patients with proliferative diabetic retinopathy, a decline in VEGF levels with active neovascularization due to anti-VEGF treatment may inhibit angiogenesis and promote fibrosis driven by connective tissue growth factor [22]. In patients with ROP, there are several reports of vitreoretinal traction band formation and retinal detachment following anti-VEGF therapy [7, 8, 21, 23]. In our cases, the deteriorated eyes of infants 1, 3, 4, 5, and 8 that eventually progressed to stage 4 or 5 all had EFP and vitreous or/and preretinal hemorrhages before IVR. EFP combined with vitreous or preretinal hemorrhages may be an indication of poor prognosis of anti-VEGF treatment for ROP.

Vitreous or preretinal hemorrhages are other major ocular complications associated with IVR. Vitreous or preretinal hemorrhages were observed in 8% of the eyes after IVB, and all were absorbed after a few weeks [24]. Infant 6 in our series had preretinal hemorrhages in her left eye 63 days post-IVR due to the recurrence of ROP. Preretinal hemorrhages were eventually resolved. Infant 7 had preretinal hemorrhages in his right eye nine days post-IVR, and hemorrhages aggravated and expanded covering the macula. Thus, both recurrences of ROP and IVR itself can cause vitreous or preretinal hemorrhages. Any vitreal-retinal tractive force or vascular contractive force exerted on neovascularization could lead to bleeding.

The limitations of this study include its retrospective nature, the small-size cohort, and the varied follow-up time in a number of patients. We still have questions to be answered, regarding the optimal dosing, timing, indications, and prognostic factors of IVR treatment. Further studies are urgently needed to provide evidence-based answers to these questions.

In conclusion, our study demonstrated that contralateral eyes with ROP can take a significantly different clinical course after IVR, which is very rare in patients treated with laser [5, 25]. The high rate of reactivation is another concern that ophthalmologists should pay attention to. The use of anti-VEGF agents causes the outcome of treatment of ROP to be unpredictable with no consensus on the safety, indications, suitable timing, and doses. Weekly or even tighter follow-up schedule is required to detect vitreoretinal traction band formation and retinal detachment in time.

## Figures and Tables

**Table 1:** Patient characteristics of infants treated had different outcomes in two eyes.

Cases/gender/ eye	GA (weeks)	Birth weight (g)	Zone	Stage	Plus	PMA, weeks at IVR	First appearance of different outcome post-IVR (days)	Clinical course	PMA, weeks at laser treatment	Surgery	Final retinal reattachment	Follow-up (months)
1/M/OD	31	1350	I	3	Yes	37	22	Regression first; then recurrence as zone II stage 2 at 42 weeks PMA	43	—	y	28
1/M/OS	31	1350	I	3	Yes	37	22	Marked posterior fibrosis; then S4B at 42 weeks PMA; S5 at 43 weeks PMA	43	LSV at 45 weeks PMA	Partial reattachment	28
2/M/OD	29	1200	I	3	Yes	37	10	Regression first; then recurrence as zone II stage 2 at 43 weeks PMA	43	—	y	30
2/M/OS	29	1200	I	3	Yes	37	10	S5 with marked posterior fibrosis and VH	—	Lensectomy and vitrectomy at 57 weeks PMA	n	30
3/F/OD	30	1100	I	3	Yes	35	27	Regression first; then recurrence as zone II stage 2 at 40 weeks PMA	41	—	y	10
3/F/OS	30	1100	I	3	Yes	35	27	Marked posterior preretinal hemorrhage at 38 weeks PMA; then S4B at 56 weeks PMA	41	LSV at 58 weeks PMA	y	10
4/M/OD	28	1000	I	3	Yes	36	18	Regression first; then recurrence as zone II stage 2 at 47 weeks PMA	47	—	y	29
4/M/OS	28	1000	I	3	Yes	36	18	Marked posterior fibrosis and VH then S5 at 47 weeks PMA	47	Lensectomy at 65 weeks PMA, vitrectomy at 104 weeks PMA	n	29
5/M/OD	27	980	I	3	Yes	36	116	Regression of ROP but persistent zone II avascularity	43	—	y	18
5/M/OS	27	980	I	3	Yes	36	116	Regression of ROP but persistent zone II avascularity; then S4A at 52 weeks PMA	43	LSV at 53 weeks PMA	y	18
6/F/OD	27	1100	I	3	Yes	34	63	Regression first; then recurrence as zone II stage 1 at 43 weeks PMA	43	—	y	9
6/F/OS	27	1100	I	3	Yes	34	63	Regression first; then recurrence as zone II stage 2 and severe VH at 43 weeks PMA	43	—	y	9
7/M/OD	31	1380	I	3	Yes	42	9	Regression first; then VH appeared at 43 weeks PMA and continued to progress	43	LSV at 47 weeks PMA	y	8
7/M/OS	31	1380	I	3	Yes	42	9	Regression of ROP but persistent zone II avascularity	43	—	y	8
8/F/OD	31	1200	APROP		Yes	36	22	Regression of ROP	—	—	y	8
8/F/OS	31	1200	APROP		Yes	36	22	Plus regressed but VH progressed to fibrosis at 40 weeks PMA and TRD at 42 weeks PMA	—	LSV at 52 weeks PMA	y	8
9/F/OD	32	1690	II	3	Yes	40	4	Plus sign regressed but peripheral fibrosis progressed and then stage 4A at 42 weeks PMA	41	LSV at 42 weeks PMA	y	11
9/F/OS	32	1690	II	3	Yes	40	4	Regression of ROP but persistent zone II avascularity	41	—	y	11

APROP: aggressive posterior retinopathy of prematurity; F: female; GA: gestational age; LSV: lens-sparing vitrectomy; M: male; OD: right eye; OS: left eye; PMA: postmenstrual age; TRD: tractional retinal detachment; VH: vitreous hemorrhage.

**Table 2:** Characteristics compared with infants between asymmetric and symmetric outcome.

	Asymmetric outcome group	Symmetric outcome group	*P* value (independent *t*-test)
Number of patients (eyes)	9 (18)	75 (150)	
Gestational age at birth (weeks)	29.6 ± 1.8	29.4 ± 2.1	0.556
Birth weight (g)	1222.2 ± 216.6	1412.2 ± 335.6	0.001
PNA at IVR (days)	52.1 ± 13.2	45.5 ± 13.8	0.946
PMA at IVR (weeks)	37.0 ± 2.4	35.9 ± 2.3	0.707

PMA: postmenstrual age; PNA: postnatal age; IVR: intravitreal injection of ranibizumab.
